# Supercapsular percutaneously-assisted total hip (SuperPath) versus posterolateral total hip arthroplasty in bilateral osteonecrosis of the femoral head: a pilot clinical trial

**DOI:** 10.1186/s12891-019-3023-0

**Published:** 2019-12-31

**Authors:** Weikun Meng, Zhong Huang, Haoyang Wang, Duan Wang, Zeyu Luo, Yang Bai, Liang Gao, Guanglin Wang, Zongke Zhou

**Affiliations:** 1 Department of Orthopaedics, West China Hospital, West China School of Medicine, Sichuan University, No. 37, Wuhou Guoxue Road, 610041 Chengdu, Sichuan People’s Republic of China; 2 Center of Experimental Orthopaedics, Saarland University Medical Center, Kirrberger Strasse, Building 37, D-66421 Homburg, Saarland Germany; 3 Sino Euro Orthopaedics Network, Homburg, Saarland Germany; 4 Hannover Medical School, Institute of Neuroanatomy and Cell Biology, Hannover, Germany; 5 Center for Systems Neuroscience (ZSN), Hannover, Germany; 6 Department of Immunization, Yunnan Center for Disease Control and Prevention, Kunming, Yunnan People’s Republic of China

**Keywords:** Osteonecrosis of the femoral head (ONFH), Total hip arthroplasty, Posterolateral approach, Supercapsular percutaneously-assisted total hip arthroplasty (SuperPath), Minimal invasive surgery, Staged surgery

## Abstract

**Background:**

The supercapsular percutaneously-assisted total hip arthroplasty (SuperPath) was proposed to be minimally invasive and tissue sparing with possible superior postoperative outcomes to traditional approaches of total hip arthroplasty (THA). Here, we compared the short-term outcomes of staged THA with the SuperPath or through posterolateral approach (PLA) for bilateral osteonecrosis of the femoral head (ONFH).

**Methods:**

Patients with bilateral late-stage ONFH were prospectively recruited from our department from March 2017 to March 2018. Staged bilateral THAs with one side SuperPath and the other side PLA were performed consecutively in the same patients with right and left hips alternating within approaches. The average time interval between the staged THAs was 3 months. Perioperative status (operation time, incision length, intraoperative blood loss, soft tissue damage, and length of hospital stay) and postoperative function (range of motion, pain, and hip function) were recorded and compared between the SuperPath and PLA approaches within 12-month postoperatively.

**Results:**

Four male patients (age, 51.00 ± 4.54; BMI, 21.49 ± 1.73) with bilateral alcohol-induced ONFH (Ficat III/IV) were followed up over 12 months postoperatively. Compared with the PLA, the SuperPath yielded shorter incision length (7.62 vs. 11.12 cm), longer operation time (103.25 vs. 66.50 min), more blood loss (1108.50 vs. 843.50 ml), deficient abduction angle of the acetabular cup (38.75° vs. 44.50°), and inferior early-term hip function (Harris hip score, 72.50 vs. 83.25) at 12-month postoperatively. Soft tissue damage, length of hospital stay, postoperative pain, postoperative range of motion, and 12-month patient satisfaction were comparable between both approaches.

**Conclusion:**

The SuperPath may be a minimally invasive technique but the present study shows less favorable short-term outcomes than PLA for total hip arthroplasty in osteonecrosis of the femoral head. More investigations are required to provide convincing favorable evidences of the SuperPath over other traditional THA approaches.

**Trial registration information:**

The trial was retrospectively registered in https://www.researchregistry.com (No. Researchregistry4993) on July 04, 2019. The first participant was enrolled on March 13, 2017.

## Background

Osteonecrosis of the femoral head (ONFH), a devastating morbidity mainly in mid-aged population, usually progresses to femoral head collapse and requires a total hip arthroplasty (THA) [1]. In Asia, ONFH accounts for approximately 50% of all THA surgeries performed annually [2–4], and the THA is effectual to improve the quality of life for patients suffering from end-stage ONFH [5]. The traditional posterolateral approach (PLA) is the most widely applied approach with excellent exposure for both primary and revision hip arthroplasty [6, 7]. However, previous studies reported high risks of postoperative dislocations and periprosthetic fractures associated with the posterior approach possibly due to the extensive intraoperative impairment of periarticular soft tissues, particularly the external rotators and joint capsule [8–11].

To minimize the overall surgical aggression, the supercapsular percutaneously-assisted total hip arthroplasty (SuperPath) was proposed as an emerging minimally invasive and tissue sparing surgical technique [12]. This portal-assisted approach accesses the hip capsule superiorly through the interval between the gluteus medius and piriformis without dissecting any muscles or tendons [13, 14]. Available case series supported the SuperPath with encouraging postoperative outcomes, in terms of length of hospital stay (LOS), postoperative pain, range of motion (ROM), and recovery after surgery [12, 15, 16]. Despite the increasing clinical attention and utilization of the Superpath, outcome comparisons between the SuperPath and other traditional approaches (e.g. PLA) for THA was seldomly undertaken to specify convincing evidence of clinical benefits of this novel technique.

The present pilot study is aimed to compare the short-term outcomes of staged THA with the SuperPath and PLA for bilateral ONFH patients. We hypothesized that the SuperPath would yield superior outcomes over the PLA in terms of both the perioperative status (operation time, incision length, intraoperative blood loss, soft tissue damage, and length of hospital stay) and postoperative function (range of motion, pain, and hip function).

## Materials and methods

### Patients

This research was approved by the Medical Ethics Committee of the West China Hospital, Sichuan University, Sichuan, China. Patients with bilateral ONFH was recruited from our department from March 2017 to March 2018. Subjects included were: (1) adult surgical candidates of bilateral THA for ONFH, (2) signed consent for implanting, and (3) ability to complete scheduled postoperative 12 months follow-ups. Subject excluded were: those with non-inflammatory degenerative joint diseases (e.g. osteoarthritis and posttraumatic arthritis), inflammatory joint diseases (e.g. reactive arthritis, ankylosing spondylitis, rheumatoid arthritis, and gout), inadequate neuromuscular status (e.g. prior paralysis and inadequate abductor strength), and overt infections or distant foci of infections.

### Surgical approach

Operations were performed by a senior surgeon specialized in traumatology and lower limb reconstruction with over 15-year experience performing primary and revision THAs with the posterior approach (over 250 cases annually). The surgeon also has accomplished more than 50 SuperPath cases. Each patient underwent bilateral staged THA with one side SuperPath and the other side PLA with an average interval of 3 months, allowing for a compensation of the possible impact of the first operation on the second one (and vice versa). Both approaches were randomizedly selected for the first operation, using a shuffled deck of cards (even – SuperPath; odd – PLA) and performed in either right or left hip. Double-blindness was undertaken in the present study in which the specific approach type was unknown to both the patients and examiners (Z.H., D.W., and Y.B.) assessing patients’ outcomes. The SuperPath was performed with specific prostheses (Microport Orthopedics, Arlington, TN, USA) as described by Chow et al. [17], and the PLA was accomplished with prostheses (DePuy Synthes, Warsaw, IN, USA) as described by Moore AT et al. [6].

Preoperative data were collected for each subject, including the age, gender, etiology, age of pain onset, history of hip injury/surgery, BMI, occupation category [18], American Society of Anesthesiologists (ASA) score [19], and Ficat stage [20]. Operation time was recorded from the initiation of incision to end of closure, and incision length was approximated with the linen tape along the surgical incision. The LOS, transfusion, complications, and readmission were also recorded.

Standardized patient care was provided including infection prophylaxis, venous thromboembolism prevention, nausea and vomiting management, wound care, and functional rehabilitation.

### Postoperative rehabilitation

Identical rehabilitation program was undertaken for all patients after both SuperPath and PLA. Briefly, Immediate hip flexion, pneumatic compression with foot pumps, and deep breathing exercise were emphasized to minimize thromboembolic and pulmonary complications. After obtaining approvals from the physical therapists, patients began indoor walking independently using crutches with tolerated weight-bearing. Self-care and home-based rehabilitation were educated before discharge, in which patients were instructed to daily walk and gradually increase the walking distance towards a goal of 2 km. All patients were generally discharged and allowed for walking with a cane on the postoperative day 3.

### Perioperative total blood loss

Perioperative total blood loss was indirectly calculated from the change in the hematocrit (Hct) according to the Gross formula [21]:

Total blood loss = PBV × (Hct_pre_ - Hct_post_) / Hct_ave_.

where Hct_pre_ is the initial preoperative Hct, Hct_post_ is the Hct on the morning of the postoperative day 3, and Hct_ave_ is the average of the Hct_pre_ and Hct_post_.

The patient’s blood volume (PBV, mL) was estimated according to the Nadler formula [22]:

PBV = k1 × height (m) + k2 × weight (kg) + k3.

where k1 = 0.3669, k2 = 0.03219, and k3 = 0.6041 for males; and k1 = 0.3561, k2 = 0.03308, and k3 = 0.1833 for females.

### Perioperative serum markers

Serum markers are widely used to evaluate soft tissue damage in the hip arthroplasty [23–27] and mainly include the creatine kinase (CK), C-reactive protein (CRP), and erythrocyte sedimentation rate (ESR). Levels of these serum markers were recorded for each patient on the day of hospital admission, postoperative day 1, day 3, and day 14, respectively.

### Acetabular component positioning analysis

Standardized anteroposterior pelvic radiographs were acquired on the postoperative day 1. Inclination and anteversion angles were measured with a computer-assisted measurement system (Japan Medical Material, Osaka, Japan). Concisely, an ellipse was fitted to the rim of the acetabular shell on radiographs. Inclination angle was defined as the angle between the longitudinal axis of the body and the acetabular axis [28]. Anteversion angle was defined with the ratio between the lengths of the minor and major axes of the ellipse [29].

### Pain, range of motion, hip function, patient satisfaction

The patient reported pain was measured with a visual analogue scale from 0 (no pain) to 10 (worst imaginable pain) at the day of hospital admission, postoperative day 1, day 3, day 14, 3 months, 6 months, and 12 months, respectively [30]. The ROMs was measured using a goniometer at the day before surgery, postoperative 3 months, 6 months, and 12 months [31]. The Harris hip score (HHS) was determined for each patient at the day of hospital admission, postoperative day 1, day 3, day 14, 3 months, 6 months, and 12 months, respectively [32]. Patient satisfaction was recorded based on the dichotomous responses (satisfied or unsatisfied) of each patient at the postoperative 3 months [33].

### Statistical analysis

Values are expressed as mean ± standard deviation. One-way ANOVA was performed to compare the VAS and HHS between the different assessment timepoints.

## Results

### Patient demographics and surgical details

Four middle-aged (mean, 51 years old; range, 45–56 years) male patients was included with a mean BMI 21.49 kg/m^2^ (range, 19.60–23.04 kg/m^2^) (Table 1). All patients were diagnosed as bilateral ONFH (Ficat stage III or IV) induced by alcohol abuse and without history of hip injury/surgery.

**Table 1:** Demographic characteristics of patients

Parameters	SuperPath	PLA
Age (years)	51.00 ± 4.54	51.00 ± 4.54
Gender (%)	Male (100%)	Male (100%)
BMI (kg/m^2^)	21.49 ± 1.73	21.49 ± 1.73
Etiology	Alcohol abuse	Alcohol abuse
History of hip injury	n.a.	n.a.
History of hip surgery	n.a.	n.a.
ASA grade	1.66 ± 0.58	1.66 ± 0.58
Age of pain onset (years)		
Left	3.00 ± 1.41	2.50 ± 0.71
Right	3.50 ± 2.12	2.50 ± 2.12
Ficat stage		
III	2	1
IV	2	3
Surgical side		
Left	2	2
Right	2	2
Postoperative complication	n.a.	n.a.
Readmission	n.a.	n.a.

*ASA* American Society of Anesthesiologists, *BMI* Body mass index, *ONFH* Osteonecrosis of the femoral head, *PLA* Posterolateral approach, *SuperPath* Supercapsular Percutaneously-Assisted Total Hip, *L* Left hip, *R* Right hip, *n.a.* Not applicable

**Fig. 1:**
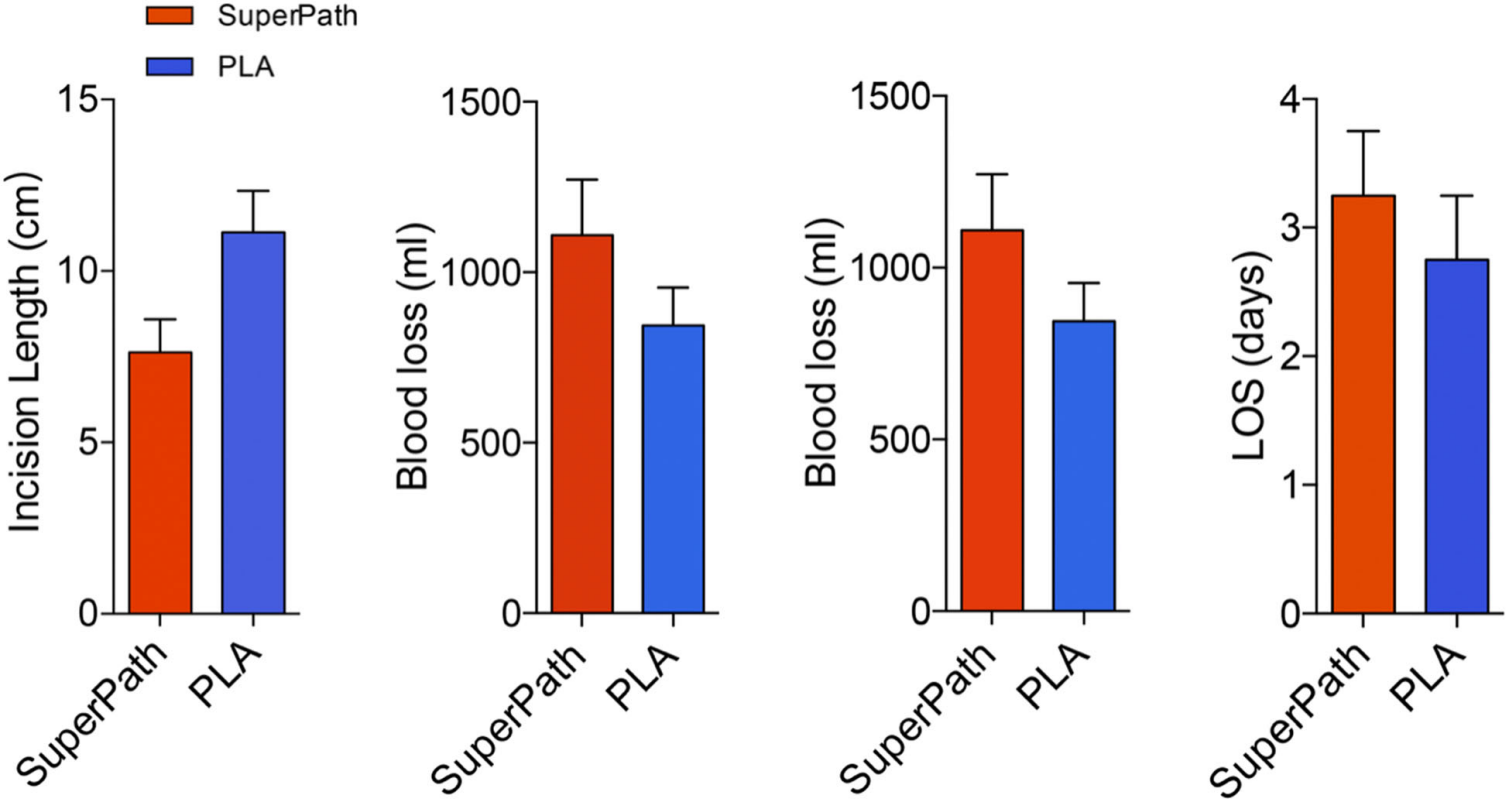
Comparisons of perioperative data. Compared with the PLA approach, the SuperPath approach was associated with relatively shorter incision length, but drastically longer operation time and more total blood loss. Patients of both approaches obtained a comparable length of hospital stay

The incision length in the SuperPath approach (7.62 ± 0.97 cm) was shorter than the PLA approach (11.12 ± 1.21 cm) (Fig. 1). However, the SuperPath approach was associated with a longer operation time (103.25 ± 12.41 min) than the PLA approach (66.50 ± 13.79 min) (Table 2). The mean blood loss was also higher in the SuperPath approach (1108.50 ml) than in the PLA approach (843.50 ml). Patients of both approaches received no blood transfusion and had a comparable length of hospital stay (SuperPath, 3.25 ± 0.50 days, PLA, 2.75 ± 0.50 days). No postoperative complications (e.g. dislocation, periprosthetic fracture, and periprosthetic joint infection) and readmission were reported within the 12 months follow-up.

**Table 2:** Perioperative data

Parameters	SuperPath	PLA
Operation time (mins)	103.25 ± 12.41	66.50 ± 13.79
Incision length (cm)	7.62 ± 0.97	11.12 ± 1.21
Blood loss (ml)	1108.50 ± 163.63	843.50 ± 111.60
Transfusion	0	0
Length of stay (days)	3.25 ± 0.50	2.75 ± 0.50

Values are expressed as mean ± standard deviation. *PLA* Posterolateral approach, *SuperPath* Supercapsular percutaneously-assisted total hip arthroplasty, *n.a.* Not applicable

### Perioperative serum markers change

**Fig. 2:**
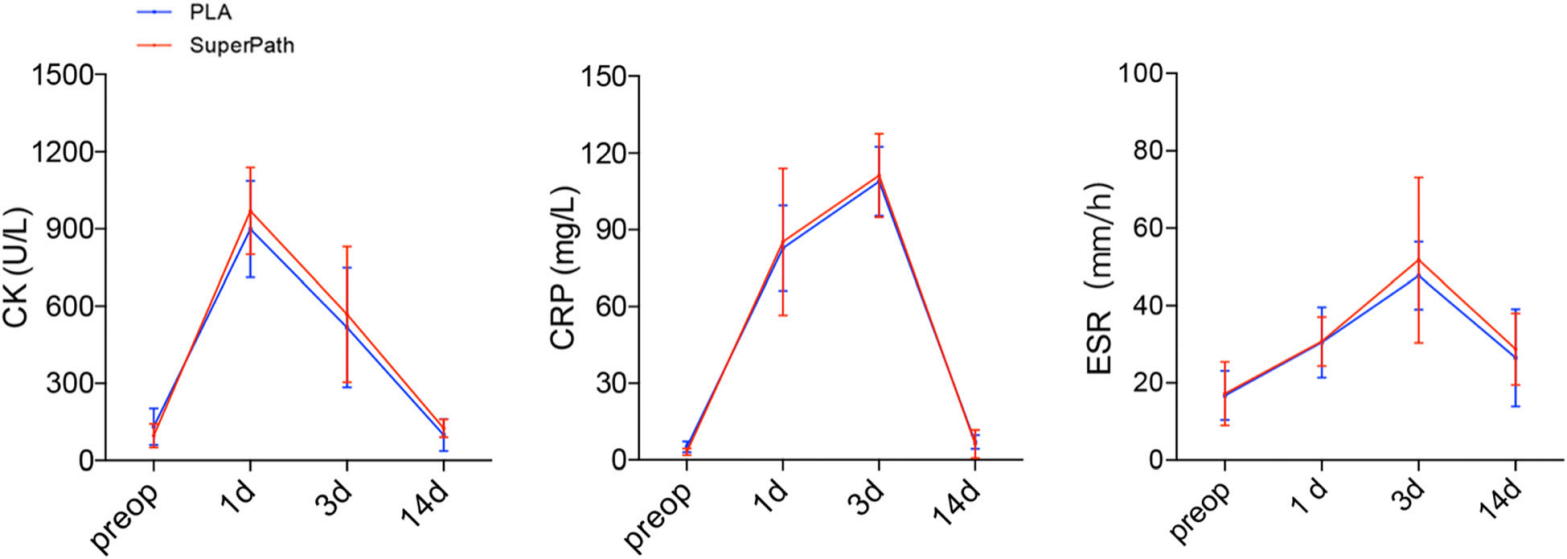
Perioperative serum markers change. Levels of serum CK, CRP, and ESR remained relatively higher in the SuperPath approach than in the PLA approach at each timing. Both CRP and CK reached their maximum at the postoperative day 3, while the ESR increased to the maximum at the postoperative day 1

The serum markers, including CRP, CK, and ESR, showed equivalent trends in both approaches within 2 weeks postoperatively (Fig. 2). Levels of all serum markers remained relatively higher in the SuperPath approach than in the PLA approach at each timing (Additional file 1: Table S1). Specially, both CK and CRP reached the maximal levels (SuperPath, 970.25 U/L, PLA, 899.50 U/L; SuperPath,111.15 mg/L, PLA,108.87 mg/L, respectively) at the postoperative day 3, while the ESR increased to the maximal level (SuperPath, 51.75 mm/h; PLA, 47.75 mm/h) at the postoperative day 1.

### Acetabular cup position

Postoperative radiographs showed that the cup abduction angle was lower in the SuperPath approach (38.75°) than in the PLA approach (44.50°) (Table 3; Additional file 1: Table S2). The average cup anteversion angle was comparable between the SuperPath (15.00°) and PLA (14.25°) approaches.

**Table 3:** Radiologic evaluation of the acetabular cup positioning

Parameters	SuperPath	PLA
Abduction angle (degrees)	38.75 ± 8.21	44.50 ± 3.64
Anteversion angle (degrees)	15.00 ± 1.82	14.25 ± 2.06

Values are expressed as mean ± standard deviation. *PLA* Posterolateral approach, *SuperPath* Supercapsular percutaneously-assisted total hip arthroplasty

### Range of motion

**Fig. 3:**
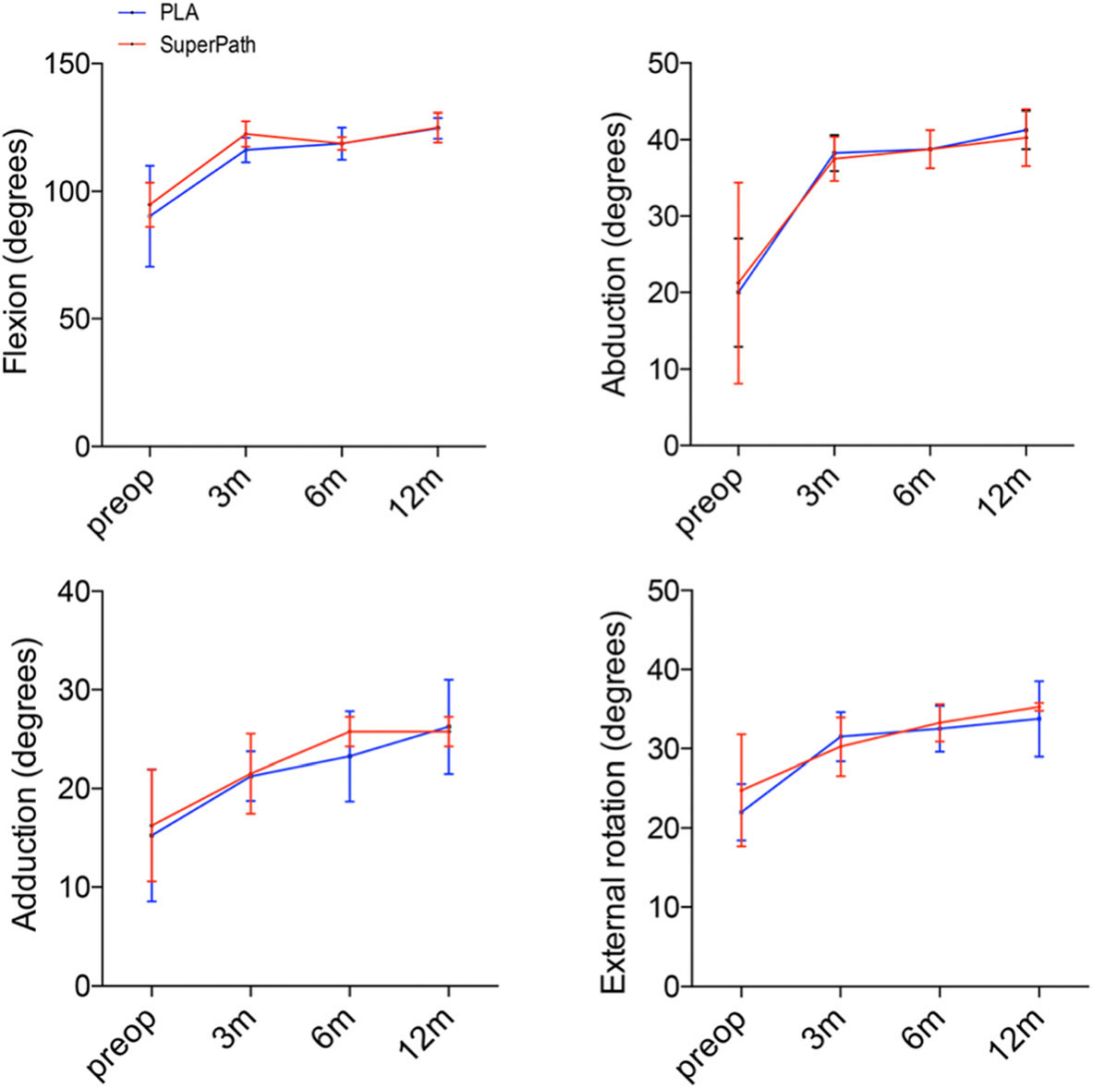
Range of motion

The ROM of hips was improved in both approaches compared with the baselines (Additional file 1: Table S3). Specifically, the hip flexion in both Superpath and PLA approaches were increased considerably from 94.75° and 90.25° preoperatively to 125.00° and 124.75° at 12 months postoperatively, respectively (Fig. 3). The hip abduction was also improved notably in both approaches (Superpath, 40.25°; PLA, 41.25°) at 12 months postoperatively. Likewise, the hip adduction and external rotation were increased appreciably in both approaches at 12 months postoperatively. However, no differences of range of motion were identified between the two approaches at each timing within the postoperative 12 months.

### Pain, hip function, and patient satisfaction

**Fig. 4:**
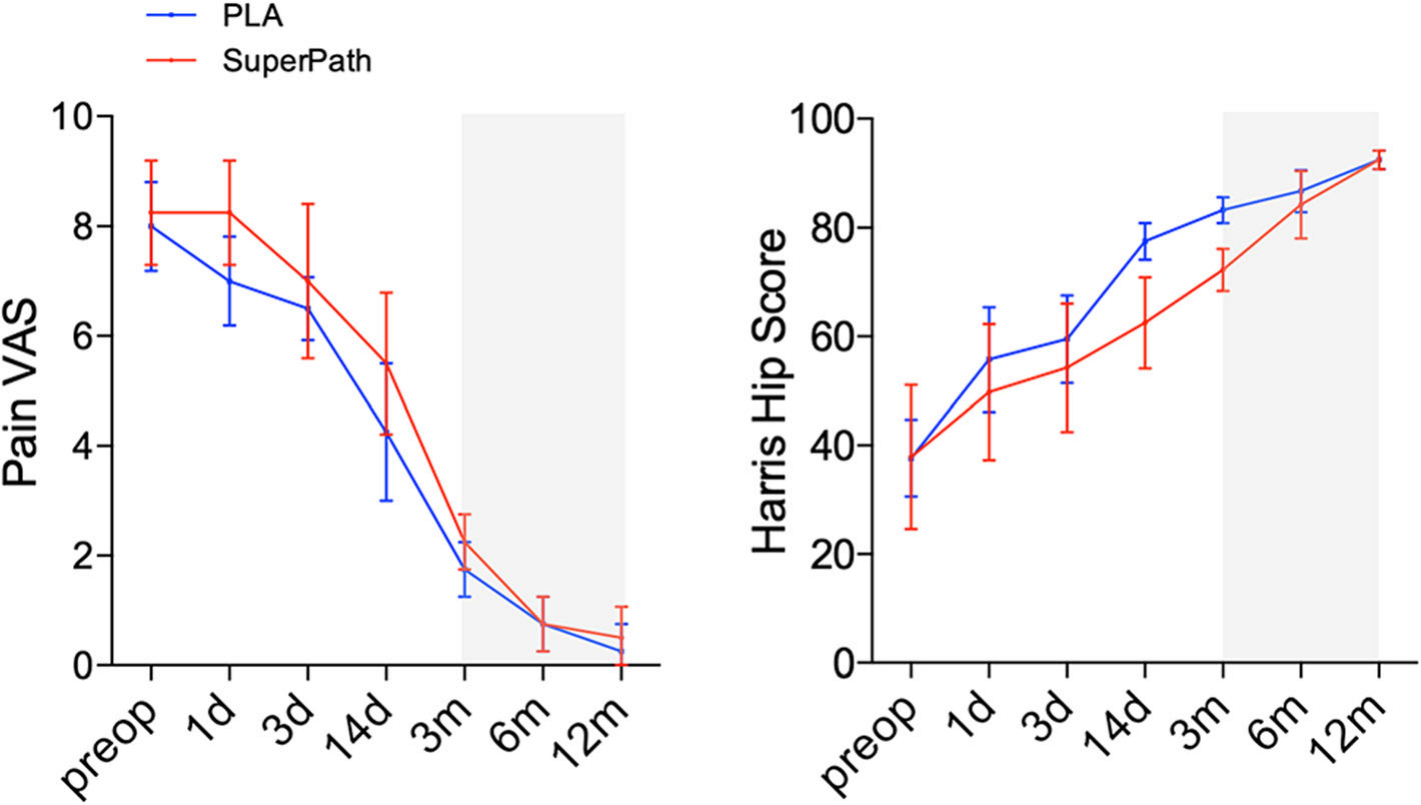
Pain VAS and Harris Hip Score. The grey zones indicate the improvements of both the postoperative VAS and Harris Hip Score reach the minimum and maximum plateaus, respectively, between postoperative 3 months and 12 months in both approaches

The mean pain VAS of the SuperPath and PLA approaches were decreased from 8.25 and 8.00 preoperatively to 0.50 and 0.25 at 12 months postoperatively, respectively (Fig. 4; Table 4; Additional file 1: Table S3). Interestingly, the pain VAS improved considerably at the postop day 14 following the SuperPath approach and at the postop day 3 following the PLA approach, however, it reached the minimum plateau between 3 months and 12 months in both approaches. Similarly, the HHSs were remarkably increased in both approaches (Superpath, 92.50; PLA, 92.50) at 12 months postoperatively compared with the preop baselines (Fig. 4; Table 4; Additional file 1: Table S4). Intergroup comparisons showed that the PLA achieved an improved hip function over the SuperPath with 15-point and 11-point increases at the postoperative day 14 and 3 months, respectively (Additional file 1: Table S3). Of note, the hip function improved noticeably after postop 3 months in the SuperPath approach and after postop day 3 in the PLA approach, however, such an improvement reached the maximal plateau between 3 months and 12 months in both approaches (Additional file 1: Table S4). Moreover, the dichotomous patients’ satisfactory score revealed that more patients were satisfied with the PLA (75%) rather than the SuperPath approach (25%) (Table 5).

**Table 4:** Pain VAS and HHS

Parameters	Timings	SuperPath	PLA
Pain VAS	preop	8.25 ± 0.95	8.00 ± 0.81
	postop day 1	8.25 ± 0.95	7.00 ± 0.81
	postop day 3	7.00 ± 1.41	6.50 ± 0.57
	postop day 14	5.50 ± 1.29	4.25 ± 1.25
	postop 3 months	2.25 ± 0.50	1.75 ± 0.50
	postop 6 months	0.75 ± 0.50	0.75 ± 0.50
	postop 12 months	0.50 ± 0.57	0.25 ± 0.50
HHS	preoperative	37.86 ± 13.27	37.66 ± 7.02
	postop day 1	49,75 ± 12.50	55.75 ± 9.60
	postop day 3	54.25 ± 11.79	59.50 ± 8.06
	postop day 14	62.50 ± 8.34	77.50 ± 3.41
	postop 3 months	72.25 ± 3.86	83.25 ± 2.36
	postop 6 months	84.25 ± 6.18	86.75 ± 3.86
	postop 12 months	92.50 ± 1.73	92.50 ± 1.73

Values are expressed as mean ± standard deviation. *HHS* Harris hip score, *PLA* posterolateral approach, *SuperPath* Supercapsular Percutaneously-Assisted Total Hip, *VAS* Visual analogue scale, *n.a.* Not applicable

**Table 5:** Satisfaction of patients

	SuperPath	PLA
Satisfaction	1	3
Non-Satisfaction	3	1

*PLA* Posterolateral approach, *SuperPath* Supercapsular Percutaneously-Assisted Total Hip

## Discussion

The present study identified that the SuperPath yielded relatively longer operation time, more total blood loss, deficient acetabular cup positioning, and inferior early-term hip function compared with the traditional posterolateral approach for total hip arthroplasty within 12-month postoperatively. However, the difference of soft tissue damage, length of hospital stay, postoperative pain, range of motion, and patient satisfaction were comparable between both approaches.

The definition of “a minimal invasive surgery” is still under debate, and the SuperPath might not be faithfully minimal invasive with more advantages than traditional approaches (e.g., the PLA) for total hip arthroplasty. Rachbauer and colleagues defined “a minimal invasive surgery” with the following characteristics: a short skin incision, preventing muscle splitting and/or detachment, and preserving the joint capsule [34]. Previous studies claimed that the SuperPath, approach as a true tissue-sparing minimally invasive approach, has less muscle damage mainly due to the preservation of external rotators [12, 15, 16, 35–37]. Our data discovered indeed a considerably shorter incision length in the SuperPath approach, however, and identified a noticeably longer operation time, more intraoperative blood loss, and comparable extents of soft tissue damage in the SuperPath approach compared with the PLA approach within the first 2 weeks postoperatively. Such unexpected outcomes with a trend towards lower patient satisfaction in the SuperPath approach are possibly attributed to the intraoperative mechanical stresses from the specific trocar cannula [38] and the elongated operation time [39–41].

Acetabular cup positioning impacts wear rates and long-term stability of the prostheses [42–44]. Biedermann et al. reported that hips dislocated posteriorly were less abducted than non-dislocating hips following the anterolateral approach and identified a statistically significant reduced dislocation risk from cups with 35°–55° abduction angle following the transgluteal approach [45]. The present study identified a relatively lower average abduction angle in the SuperPath approach (38.75°) than in the PLA approach (44.50°), possibly hinting an increased dislocation risk in the long-term. However, these data were analyzed with the only one available clinical trial from Xie et al., comparing the SuperPath and PLA for THA, which reported comparable abduction angles of 43.60° and 44.50° for both approaches [37]. Such a disagreement might be attributed to the relatively poorer exposure during the SuperPath procedure, hindering a proper intraoperative positioning of the acetabular component. Therefore, further studies with a greater sample size and longer follow-up might illustrate the possible variance of hip stability after both approaches.

Interestingly, the pain VAS following the SuperPath approach improved slower than that following the PLA approach, and both approaches reached the comparable levels between 3 months and 12 months postoperatively, as well as the equivalent range of motion at 12 months postoperatively. These data also contradict with previous encouraging outcomes from the above-mentioned clinical trial from Xie and colleagues [37]. Noteworthily, the average operation time of PLA in Xie’s study was 106.5 min (range, 90–133 min), which was considerably shorter than the previous studies with a mean operation time within 60 min [46, 47]. Therefore, the encouraging outcomes of the SuperPath approach are possibly attributed to their dissatisfied performance in the PLA controls.

Remarkably, the HHS demonstrated relatively impaired early-term hip function in the SuperPath approach than in the PLA approach. The improvement of hip function after the SuperPath approach is slower than that after the PLA approach, and the HHS was 15-point (at the postoperative day 14) and 11-point (at the postoperative 3 months) lower in the SuperPath approach compared with the PLA approach and gradually approached comparable levels after 6 months postoperatively. One possible explanation of such impaired early-term hip function in the SuperPath approach might be the longer operation time and more total blood loss, which were previously reported as major factors influencing the functional recovery and quality of life of patients [48, 49].

Moreover, the learning curve of new technically demanding techniques should not be underestimated, which is defined as the number of times an approach must be repeated before reaching a steady plateau. Compared with other approaches, the SuperPath is more subtle with changes in implant and instrument design and requires surgeons to augment performances via the fine-tuning with the learning curve [37]. The only clinical study has compared learning curves of the percutaneously assisted total hip arthroplasty (PATH) and SuperPath in terms of operation time [36]. However, a precise “learning curve” ought to be established in terms of short- and long-term outcomes instead of merely the operation time [50–52]. Hence, more randomized controlled trials are necessitated to define the minimum number of cases required to complete a learning curve of SuperPath.

A standardized multidisciplinary teamwork of physicians, surgeons, physical therapists, and physiologists, and patient family (caregivers) is critical to ensure an effective performance of the implanted hip prosthesis, which is generally applied to all THA patients treated in our clinic [53]. This might partially explain the long-standing complication-free status following both approaches in our cases. The early and in-depth involvement of the physical therapist is highly recommended and the rehabilitation program should be prepared individually. Vigilance from the patient family (caregivers) is also essential to ensure the implants utilize properly and to prevent implant-related complications.

Several limitations exist in the present study. Firstly, the sample size of the current study is greatly limited and the postoperative follow-up is relatively short, which might not allow us to draw a definitive conclusion of both approaches. Secondly, different hip implants were utilized in both approaches, which might influence the final postoperative outcomes. However, the staged bilateral hip arthroplasty in identical individuals with a comprehensive self-comparison between the SuperPath and PLA and the significant differences of both preoperative status and short-term postoperative function opens up new questions for further comparisons of the SuperPath with other traditional THA approaches.

## Conclusions

In summary, the SuperPath technique may be a minimally invasive technique but our data show less favorable outcomes than PLA for total hip arthroplasty in osteonecrosis of the femoral head. More randomized controlled trials are required to define the learning curve of the SuperPath technique, in terms of postoperative outcomes, and to provide convincing evidence of its clinical benefits over other traditional THA approaches.

## Supplementary information

**Additional file 1: Table S1.** Perioperative changes of serum markers. **Table S2.** Range of motion. **Table S3.** Comparisons of the range of motion, pain VAS, HHS at the day before surgery and postoperative 12 months. **Table S4.** Comparisons of the pain VAS and HHS between each time point.

## Data Availability

The datasets generated and/or analysed during the current study are not publicly available due to local data protection and confidentiality policy but are available from the corresponding author on reasonable request.

## Abbreviations

HHS: Harris Hip Score
LOS: Length of Hospital Stay
ONFH: Osteonecrosis of the Femoral Head
PLA: Posterolateral Approach
ROM: Range of Motion
SuperPath: Supercapsular Percutaneously-assisted Total Hip
THA: Total Hip Arthroplasty
