# Supplementary material for: Supercapsular percutaneously-assisted total hip (SuperPath) versus posterolateral total hip arthroplasty in bilateral osteonecrosis of the femoral head: a pilot clinical trial
Source: BMC Musculoskelet Disord. 2019 Dec 31;21:2. doi: 10.1186/s12891-019-3023-0 (PMC6937651; doi:10.1186/s12891-019-3023-0)
Supplement: Supplementary file 1 — Additional file 1: Table S1. Perioperative changes of serum markers. Table S2. Range of motion. Table S3. Comparisons of the range of motion, pain VAS, HHS at the day before surgery and postoperative 12 months. Table S4. Comparisons of the pain VAS and HHS between each time point. [file 12891_2019_3023_MOESM1_ESM.docx]

**Supplementary Materials**

**Supercapsular percutaneously-assisted total hip (SuperPath) versus posterolateral total hip arthroplasty in bilateral osteonecrosis of the femoral head: a pilot clinical trial**

Weikun Meng^1,2,3,†^, Zhong Huang^1,3,4,5,†^, Haoyang Wang^1^, Duan Wang^1^, Zeyu Luo^1^,

Yang Bai^6^, Liang Gao^2,3,^*, Guanglin Wang^1,^*, Zongke Zhou^1,^*

^1^Department of Orthopaedics, West China Hospital, West China School of Medicine, Sichuan University, Chengdu, Sichuan, P.R. China

^2^Center of Experimental Orthopaedics, Saarland University Medical Center, Homburg, Saarland, Germany

^3^Sino Euro Orthopaedics Network, Homburg, Saarland, Germany

^4^Institute of Neuroanatomy and Cell Biology, Hannover Medical School, Hannover, Germany

^5^Center for Systems Neuroscience (ZSN), Hannover, Germany

^6^Department of Immunization, Yunnan Center for Disease Control and Prevention, Kunming, Yunnan, P.R. China

†Denotes co-first authors.

*Authors to whom correspondence should be addressed:

Liang Gao, M.D., Ph.D., Center of Experimental Orthopaedics, Saarland University, Kirrberger Strasse, Building 37, D-66421 Homburg, Saarland, Germany

Tel.: +49-06841-1624569. Email: liang.gao@uni-saarland.de

Guanglin Wang, M.D., Department of Orthopaedics, West China Hospital, West China School of Medicine, Sichuan University, No. 37, Wuhou Guoxue Road, 610041 Chengdu, P. R. China

Tel.: +86-028-85422570. E-mail: wglfrank@163.com

Zongke Zhou, M.D., Department of Orthopaedics, West China Hospital, West China School of Medicine, Sichuan University, No. 37, Wuhou Guoxue Road, 610041 Chengdu, P. R. China

Tel.: +86-028-85422570. E-mail: zongke@126.com

**Table S1**. Perioperative changes of serum markers.

| **Serum markers** | **Assessment time points** | **SuperPath** | **PLA** |
| --- | --- | --- | --- |
| CRP (mg/L) | preop | 3.12 ± 1.30 | 5.07 ± 2.13 |
|  | postop day 1 | 85.25 ± 28.72 | 82.77 ± 16.67 |
|  | postop day 3 | 111.15 ± 16.33 | 108.87 ± 13.50 |
|  | postop day 14 | 6.16 ± 5.45 | 6.97 ± 2.73 |
| ESR (mm/h) | preop | 17.25 ± 8.22 | 16.75 ± 6.34 |
|  | postop day 1 | 30.75 ± 6.34 | 30.50 ± 9.11 |
|  | postop day 3 | 51.75 ± 21.42 | 47.75 ± 8.77 |
|  | postop day 14 | 28.75 ± 9.28 | 26.50 ± 12.60 |
| CK (U/L) | preop | 96.25 ± 46.34 | 131.75 ± 70.79 |
|  | postop day 1 | 970.25 ± 169.15 | 899.50 ± 188.10 |
|  | postop day 3 | 568.50 ± 263.57 | 517.25 ± 231.99 |
|  | postop day 14 | 125.25 ± 34.89 | 98.75 ± 62.41 |

Values are expressed as mean ± standard deviation. PLA, posterolateral approach; SuperPath, supercapsular percutaneously-assisted total hip arthroplasty; CRP, C-reactive protein; CK, Creatine kinase; ESR, Erythrocyte sedimentation rate.

**Table S2.** Range of motion.

| **Types of motion** | **Assessment time points** | **SuperPath** | **PLA** |
| --- | --- | --- | --- |
| Flexion | preop | 94.75 ± 8.73 | 90.25 ± 19.85 |
|  | postop 3 months | 112.50 ± 5.00 | 116.25 ± 4.78 |
|  | postop 6 months | 118.75 ± 2.50 | 118.75 ± 6.29 |
|  | postop 12 months | 125.00 ± 5.77 | 124.75 ± 4.11 |
| Abduction | preop | 21.25 ± 13.14 | 20.00 ± 7.07 |
|  | postop 3 month | 37.50 ± 2.88 | 38.25 ± 2.36 |
|  | postop 6 month | 38.75 ± 2.50 | 38.75 ± 2.50 |
|  | postop 12 months | 40.25 ± 3.68 | 41.25 ± 2.50 |
| Adduction | preop | 16.25 ± 5.67 | 15.25 ± 6.70 |
|  | postop 3 months | 21.50 ± 4.04 | 21.25 ± 2.50 |
|  | postop 6 months | 25.75 ± 1.50 | 23.25 ± 4.57 |
|  | postop 12 months | 25.75 ± 1.50 | 26.25 ± 4.78 |
| External rotation | preop | 24.75 ± 7.08 | 22.00 ± 3.55 |
|  | postop 3 months | 30.25 ± 3.68 | 31.50 ± 3.10 |
|  | postop 6 months | 33.25 ± 2.36 | 32.50 ± 2.88 |
|  | postop 12 months | 35.25 ± 0.50 | 33.75 ± 4.78 |

Values are expressed as mean ± standard deviation. PLA, posterolateral approach; SuperPath, Supercapsular Percutaneously-Assisted Total Hip.

**Table S3**. Comparisons of the range of motion, pain VAS, HHS at the day before surgery and postoperative 12 months.

| **Parameters** | **SuperPath** | |  | **PLA** | |
| --- | --- | --- | --- | --- | --- |
|  | **Preop** | **Postop 12 months** |  | **Preop** | **Postop 12 months** |
| Flexion | 94.75 ± 8.73 | 125.00 ± 5.77 |  | 90.25 ± 19.85 | 124.75 ± 4.11 |
| Abduction | 21.25 ± 13.14 | 40.25 ± 3.68 |  | 20.00 ± 7.07 | 41.25 ± 2.50 |
| Adduction | 16.25 ± 5.67 | 25.75 ± 1.50 |  | 15.25 ± 6.70 | 26.25 ± 4.78 |
| External rotation | 24.75 ± 7.08 | 35.25 ± 0.50 |  | 22.00 ± 3.55 | 33.75 ± 4.78 |
| Pain VAS | 8.25 ± 0.95 | 0.50 ± 0.57 |  | 8.00 ± 0.81 | 0.25 ± 0.50 |
| HHS | 37.86 ± 13.27 | 92.50 ± 1.73 |  | 37.66 ± 7.02 | 92.50 ± 1.73 |

Values are expressed as mean ± standard deviation. HHS, Harris hip score; PLA, posterolateral approach; SuperPath, Supercapsular Percutaneously-Assisted Total Hip; VAS, visual analogue scale.

**Table S4**. Comparisons of the pain VAS and HHS between each time point.

| **Comparisons between assessment time points** | **VAS** | |  | **HHS** | |
| --- | --- | --- | --- | --- | --- |
|  | **SuperPath** | **PLA** |  | **SuperPath** | **PLA** |
| Preop vs. Postop day 1 | 1.000 | 0.519 |  | 0.569 | **0.005** |
| Preop vs. Postop day 3 | 0.527 | 0.121 |  | 0.219 | **0.001** |
| Preop vs. Postop day 14 | **0.008** | **< 0.001** |  | **0.018** | **< 0.001** |
| Preop vs. Postop 3m | **< 0.001** | **< 0.001** |  | **0.001** | **< 0.001** |
| Preop vs. Postop 6m | **< 0.001** | **< 0.001** |  | **< 0.001** | **< 0.001** |
| Preop vs. Postop 12m | **< 0.001** | **< 0.001** |  | **< 0.001** | **< 0.001** |
| Postop day 1 vs. Postop day 3 | 0.527 | 0.962 |  | 0.992 | 0.969 |
| Postop day 1 vs. Postop day 14 | **0.008** | **0.001** |  | 0.478 | **0.001** |
| Postop day 1 vs. Postop 3m | **< 0.001** | **< 0.001** |  | **0.035** | **< 0.001** |
| Postop day 1 vs. Postop 6m | **< 0.001** | **< 0.001** |  | **0.001** | **< 0.001** |
| Postop day 1 vs. Postop 12m | **< 0.001** | **< 0.001** |  | **< 0.001** | **< 0.001** |
| Postop day 3 vs. Postop day 14 | 0.322 | **0.006** |  | 0.864 | **0.005** |
| Postop day 3 vs. Postop 3m | **< 0.001** | **< 0.001** |  | 0.137 | **< 0.001** |
| Postop day 3 vs. Postop 6m | **< 0.001** | **< 0.001** |  | **0.003** | **< 0.001** |
| Postop day 3 vs. Postop 12m | **< 0.001** | **< 0.001** |  | **< 0.001** | **< 0.001** |
| Postop day 14 vs. Postop 3m | **0.001** | **0.002** |  | 0.751 | 0.809 |
| Postop day 14 vs. Postop 6m | **< 0.001** | **< 0.001** |  | **0.044** | 0.332 |
| Postop day 14 vs. Postop 12m | **< 0.001** | **< 0.001** |  | **0.003** | **0.025** |
| Postop 3m vs. Postop 6m | 0.322 | 0.519 |  | 0.546 | 0.978 |
| Postop 3m vs. Postop 12m | 0.175 | 0.121 |  | 0.071 | 0.332 |
| Postop 6m vs. Postop 12m | 1.000 | 0.962 |  | 0.864 | 0.809 |

Comparisons of the pain VAS and HHS between each time point were analyzed with 0ne-way ANOVA with post-hoc Tukey HSD. All P values < 0.05 are highlighted in bold. HHS, Harris hip score; PLA, posterolateral approach; SuperPath, Supercapsular Percutaneously-Assisted Total Hip; VAS, visual analogue scale.
